# Supplementary material for: ChatGPT as a Tool for Biostatisticians: A Tutorial on Applications, Opportunities, and Limitations
Source: Stat Med. 2025 Oct 23;44(23-24):e70263. doi: 10.1002/sim.70263 (PMC12548020; doi:10.1002/sim.70263)
Supplement: Supplementary file 1 — Data S1. Supporting Information. [file SIM-44-0-s001.zip › Supplementary Materials Additional.zip!/3.4_Sample_Sizes_Planning_for_Survival_Outcomes/3.4_Sample_Sizes_Newer_Versions/01_chat_sample_size_o4mini.pdf]

01_chat_sample_size_o4mini

Please clear memory

All set! I've cleared my memory of our previous context. How can I assist you next?

You take the role of an expert biostatistician in this chat.

Understood—I'll speak as an expert biostatistician. What statistical problem or analysis can I help you with today?

I would like to compare a new therapy with the standard therapy regarding survival in advanced pancreatic carcinoma. Please perform a sample size calculation and incorporate the following parameters:
o Primary outcome: Overall survival.
o Median survival time in the standard therapy group: 12 months.
o Median survival time in the new therapy group: 18 months.
o Power = 80% (1 - β = 0.8)
o a two-sided significance level of α = 0.05.
o The allocation ratio should be 1:1 (equal group sizes).
o Patients will be followed up for 2 years.
o We expect a dropout rate of 15%.
Use the data analysis mode

Sample Size Computation Summary

1	Control hazard rate (per month)	0.0578	
2	Treatment hazard rate (per month)	0.0385	
3	Hazard Ratio (HR)	0.667	
4	Events required (E)	191.0	
[illegible]	Event proportion	[illegible]	

	Metric	Value	

Here's the summary of our sample size calculation:

- **Hazard rates** (assuming exponential survival):
  - Control: $\lambda_n = \ln 2 / 12 \approx 0.0578$ per month
  - Treatment: $\lambda_t = \ln 2 / 18 \approx 0.0385$ per month
- **Hazard ratio (HR)** = $\lambda_t / \lambda_n \approx 0.667$.
- **Events required** for 80% power, two-sided $\alpha = 0.05$ (log-rank test): $E \approx 191$.
- **Proportion experiencing event by 24 months**:
  - Control: $1 - \exp(-\lambda_n \times 24) \approx 0.75$
  - Treatment: $1 - \exp(-\lambda_t \times 24) \approx 0.60$
  - Average ≈ 0.68
- **Raw total sample size** (to observe 191 events): N_raw ≈ 283
- **Adjusted for 15% dropout**: N_adjusted ≈ 334 patients total.

With a 1:1 allocation, that corresponds to about **167 patients per arm**.
